# COVID-19 Induced Acute Respiratory Distress Syndrome—A Multicenter Observational Study

**DOI:** 10.3389/fmed.2020.599533

**Published:** 2020-12-18

**Authors:** Johannes Herrmann, Elisabeth Hannah Adam, Quirin Notz, Philipp Helmer, Michael Sonntagbauer, Peter Ungemach-Papenberg, Andreas Sanns, York Zausig, Thorsten Steinfeldt, Iuliu Torje, Benedikt Schmid, Tobias Schlesinger, Caroline Rolfes, Christian Reyher, Markus Kredel, Jan Stumpner, Alexander Brack, Thomas Wurmb, Daniel Gill-Schuster, Peter Kranke, Dirk Weismann, Hartwig Klinker, Peter Heuschmann, Viktoria Rücker, Stefan Frantz, Georg Ertl, Ralf Michael Muellenbach, Haitham Mutlak, Patrick Meybohm, Kai Zacharowski, Christopher Lotz

**Affiliations:** ^1^Department of Anesthesiology and Critical Care, University Hospital Würzburg, Julius-Maximilians-University Würzburg, Würzburg, Germany; ^2^Department of Anesthesiology, Intensive Care Medicine and Pain Therapy, University Hospital Frankfurt, Goethe-University, Frankfurt, Germany; ^3^Department of Anesthesiology and Critical Care, Klinikum Aschaffenburg-Alzenau, Aschaffenburg, Germany; ^4^Department of Anesthesiology and Critical Care, Diakoneo Diak Klinikum Schwabisch Hall, Schwabisch-Hall, Germany; ^5^Department of Critical Care, Emergency Medicine and Anesthesiology, ARDS/ECMO-Centre, Campus Kassel of the University of Southampton, Southampton, Germany; ^6^Department of Anesthesiology and Critical Care, Sana-Klinikum Offenbach GmbH, Offenbach, Germany; ^7^Department of Internal Medicine I, University Hospital Würzburg, Würzburg, Germany; ^8^Department of Internal Medicine II, University Hospital Würzburg, Würzburg, Germany; ^9^Institute for Clinical Epidemiology and Biometry, Julius-Maximilians-University, Würzburg, Germany; ^10^Clinical Trial Center, University Hospital Würzburg, Julius-Maximilians-University, Würzburg, Germany

**Keywords:** COVID-19, ARDS (acute respiratory distress syndrome), intensive care medicine, pandemia, Germany

## Abstract

**Background:** Proportions of patients dying from the coronavirus disease-19 (COVID-19) vary between different countries. We report the characteristics; clinical course and outcome of patients requiring intensive care due to COVID-19 induced acute respiratory distress syndrome (ARDS).

**Methods:** This is a retrospective, observational multicentre study in five German secondary or tertiary care hospitals. All patients consecutively admitted to the intensive care unit (ICU) in any of the participating hospitals between March 12 and May 4, 2020 with a COVID-19 induced ARDS were included.

**Results:** A total of 106 ICU patients were treated for COVID-19 induced ARDS, whereas severe ARDS was present in the majority of cases. Survival of ICU treatment was 65.0%. Median duration of ICU treatment was 11 days; median duration of mechanical ventilation was 9 days. The majority of ICU treated patients (75.5%) did not receive any antiviral or anti-inflammatory therapies. Venovenous (vv) ECMO was utilized in 16.3%. ICU triage with population-level decision making was not necessary at any time. Univariate analysis associated older age, diabetes mellitus or a higher SOFA score on admission with non-survival during ICU stay.

**Conclusions:** A high level of care adhering to standard ARDS treatments lead to a good outcome in critically ill COVID-19 patients.

## Background

Following the first outbreak of the severe acute respiratory syndrome coronavirus 2 (SARS-CoV2) in December 2019, the virus has spread worldwide. The coronavirus disease-19 (COVID-19) currently affects 188 countries and territories (1).

In Germany the first case of a SARS-CoV2 infection was diagnosed on February 27, 2020 (2). Although means of social distancing helped to contain virus transmission more than 175 000 people were infected (1). SARS-CoV2 was suggested to elicit a new ARDS-subphenotype, where hypoxemia often does not match lung compliance and ventilator responsiveness (3). The observed case-fatality ratios differ among countries, with the United States reporting 3.8% and Germany reporting 4.5%,respectively. This is lower compared to other European countries, for example, Italy (14.3%), United Kingdom (15.3%) or France (14.2%) (4). Understanding the specific characteristics of severe and fatal disease, as well as the therapeutic approaches to COVID-19 induced ARDS remains an urgent need to provide a basis for best practice models of standardized ARDS treatment.

In the current study, we report the epidemiologic features, clinical course, treatment patterns and outcome of patients requiring intensive care due to COVID-19 induced ARDS in five German centers.

## Methods

This is a retrospective, observational multicenter study at the University Hospital Würzburg and University Hospital Frankfurt, as well as the municipal hospitals of Kassel, Offenbach and Aschaffenburg. Würzburg, Frankfurt, and Kassel are referral centers for adult extracorporeal membrane oxygenation (ECMO) and part of the German ARDS network. To guarantee an individual high level of ICU care all participating hospitals immediately improved ICU infrastructure by adding extra ICU nurses, physicians, medical students and other support workers to the COVID-19 ICUs.

The institutional ethic boards of the University of Würzburg and Frankfurt, as well as the medical association of Bavaria ethics board (Aschaffenburg) and Hessen (Offenbach, Kassel), respectively, approved the study. The need for informed consent from individual patients was waived due to the context of sole retrospective chart review within standard care.

### Patient Selection

We included all patients consecutively admitted to the ICU in any of the participating hospitals due to an acute respiratory distress syndrome between March 12 and May 4, 2020. All patients submitted to the ICU had received the diagnosis of a SARS-CoV2 infection or were tested positive for COVID-19 during ICU treatment. SARS-CoV2 infection was detected with real-time reverse transcriptase polymerase chain reaction (RT-PCR) testing based on the recommended World Health Organization standards. No patient tested positive for other respiratory viruses in primary diagnostics. All patients received venous thromboembolism (VTE) prophylaxis with pharmacologic anticoagulation according to the German guidelines on VTE (5). In case of contraindications against pharmacological anticoagulation, mechanical prophylaxis (intermittent pneumatic compression) was conducted. Follow-up ended with ICU discharge or death during ICU treatment, respectively.

### Data Collection

Specific treatment protocols were not defined. Routine clinical data were continuously recorded using patient data management systems (PDMS) (University of Würzburg: COPRA6 RM1.0, COPRA System GmbH, Berlin, Germany; University of Frankfurt: Metavision 5.0, imd soft, Dusseldorf, Germany) or assessed via handwritten records (Aschaffenburg, Offenbach, Kassel). The data were retrieved according to the diagnostic standards of the individual centers. Demographic data, pre-existing medical conditions and medications were gathered from prior written records or discharge letters, questionnaires at the time of hospital admission, as well as personal communication with family members. Lung edema on chest radiographs was evaluated via the Radiographic Assessment of Lung Edema (RALE) score (6) in all patients admitted to the ICU in Würzburg. Severity of ARDS was categorized in line with the Berlin definition (mild: 200 mm Hg < PaO_2_/FIO_2_ ≤ 300 mm Hg; moderate: 100 mm Hg < PaO_2_/FIO_2_ < 200 mm Hg and severe PaO_2_/FIO_2_ < 100 mm Hg) (7). Since treatment and data acquisition were conducted according to the standard procedures of the respective hospital, diagnostics and reported parameters varied to some degree between the centers. Hence, if applicable the nominators and denominators are reported for each parameter separately, since not all parameters could be retrieved in the whole cohort of patients. All participating hospitals reported their data via a unified sheet (Microsoft® Excel 2019, Version 16.41, Microsoft® Corporation, Redmond, WA).

### Statistical Analysis

Median and interquartile range (25–75%) were reported for continuous data, absolute and relative frequencies for categorical variables. Percentages are based on the total number of patients with complete information in the respective category. Continuous variables were tested for normality using histogram and QQ-plot. To compare differences between survivors and non-survivors in continuous variables the Mann-Whitney rank-sum test or the Wilcoxon matched-pairs signed rank test, respectively, was used as appropriate, as most of the variables were not normally distributed. The Chi^2^-Test or Fisher exact test was used to assess the association of dichotomous variables and the outcome. Age-adjusted logistic regression analyses were performed to identify factors associated with death during ICU treatment. Wilson score method was used to estimate 95%-confidence intervals for the crude proportion of survival during ICU stay; Kaplan-Meier estimates were used for estimating survival probability. All tests were two-tailed, a *p*-value <0.05 was considered as statistically significant. The univariate *p*-values were based on Mann-Whitney *U* Test, Chir^2^-Test or Fisher's exact Test as appropriate. The adjusted *p*-values are based on a logistic regression adjusted for age.

Data were analyzed using SAS® Software, Version 9.4. Copyright SAS Institute Inc. Cary, NC, USA, R, R Version 3.6.2., Prism 5 for Mac OS X (GraphPad Software, San Diego, CA), Stata version 14.2 (Stata Corp, College Station, TX) or SigmaPlot®, version 10.0 (Systat Software, Erkrath, Germany).

## Results

A total of 106 ICU patients were treated for COVID-19 induced ARDS. None of these patients remained in ICU care at the end of the study period. Three patients were transferred from Italy to the ICU in Würzburg. Two of these patients were excluded from the analysis due to an advanced clinical course at the time of their transfer, as well as incomplete records and short-term ICU stay.

### Epidemiologic Characteristics and Outcome

**Figure 1:**
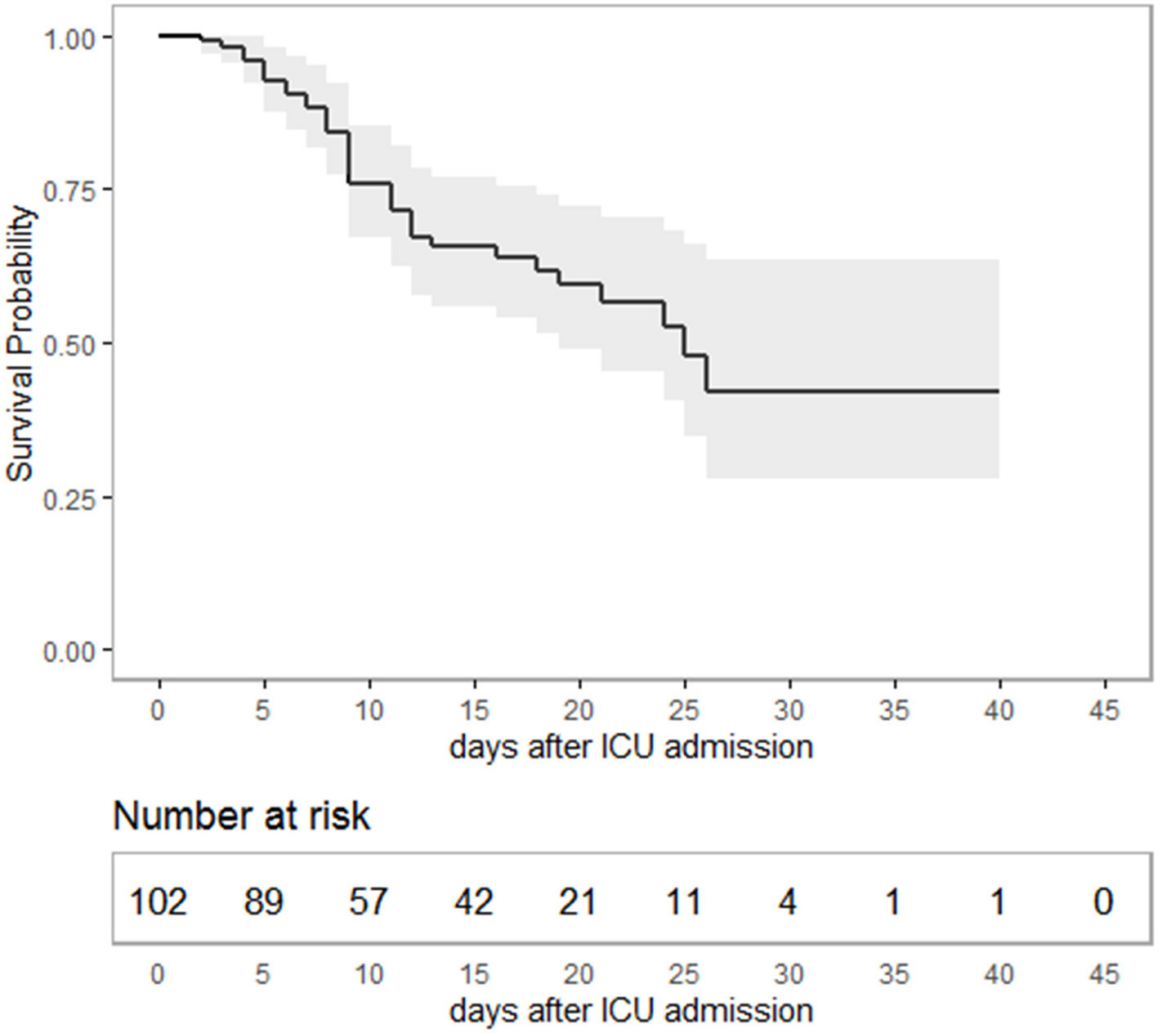
Kaplan-Meier-plot showing survival probability as a function of time in intensive care. Overall, 65% (95%-CI 55.6–73.5) of the patients survived ICU treatment with a median duration of 11 (IQR: 7–19) days. The study period ended with ICU discharge or death, respectively. Hence, survival data are terminally censored resulting in a horizontal line on the far right.

Median age of the patients was 64 (IQR 54–76) years, 70.5% were males. Median time from hospital to ICU admission was 2 (IQR 1–4) days. Overall, 37 patients died during ICU stay, constituting an overall survival of 65.0% (95% CI 55.6–73.5) (Figure 1). Considering only severe ARDS (P_a_O_2_/F_i_O_2_ < 100) (7), survival in critical care was 59.7% (CI 46.7–71.4) (Supplementary Table 1). Median duration of ICU treatment was 11 (IQR 7–19) days. Reported comorbidities were present in 79.3% of the cases, with arterial hypertension as most common comorbidity followed by diabetes mellitus (Table 1). Patients surviving ICU treatment were significantly younger. Although the majority of patients were male, a gender difference with respect to survival was not observed. Diabetes mellitus [age-adjusted Odds Ratio (OR) 3.4; 95-CI 1.3–8.7] and a higher SOFA score on admission (age-adjusted OR 1.2; 95%-CI 1.1–1.4) were associated with non-survival in univariate and age-adjusted analyses.

**Table 1:** Epidemiologic characteristics.

**Characteristic**	**All patients** **(*N* = 106)**	**Survivors** **(*N* = 69)**	**Non-survivors** **(*N* = 37)**	*P*-value^*^	**Adjusted** *p*-value^**^
**Demographics**					
Age (years)	64 (54–76)	61 (51–71)	70 (60–78)	0.0029	
Male—no. patients (%)	74(70.5)	50 (72.5)	24 (66.7)	0.5365	0.8194
BMI—median	27.8 (24.9–32.0)	27.8 (24.9–32.0)	27.4 (25.0–31.7)	0.9385	0.8029
**Co-morbidities**					
Arterial hypertension—no. patients (%)	71 (67.0)	44 (63.8)	27 (73.0)	0.3368	0.6103
Diabetes mellitus—no. patients (%)	26 (24.5)	11 (15.9)	115 (40.5)	0.0050	0.0133
COPD/asthma bronchiale—no. patients (%)	16 (15.1)	7 (10.1)	9 (24.3)	0.0519	0.1192
Coronary artery disease—no. patients (%)	20 (18.9)	9 (13.0)	11 (29.7)	0.0363	0.2236
Heart failure—No. patients (%)	15 (14.2)	7 (10.2)	8 (21.6)	0.1061	0.4755
Stroke—no. patients (%)	13 (12.3)	7 (10.1)	6 (16.2)	0.3637	0.6759
Chronic renal failure—no. patients (%)	16 (15.1)	8 (11.6)	8 (21.6)	0.1692	0.5348
Cancer—no. patients (%)	12 (11.3)	8 (11.6)	4 (10.8)	>0.900	0.3842
Duration prior to ICU admission—days in hospital	2 (0–4) (*N* = 51)	2 (0–4) (*N* = 33)	2 (1–4) (*N* = 18)	0.6519	0.9821
Body temp. > 37.5°C at time of ICU admission (%)	54 (71.0) (*N* = 76)	41 (68.9) (*N* = 52)	13 (54.2) (*N* = 24)	0.0274	0.0098
**Scores**					
SOFA at time of ICU admission	9 (4–14) (*N* = 78)	5 (4–11) (*N* = 49)	13 (9–16) (*N* = 29)	0.0002	0.0003
Highest SOFA	13 (7–18) (*N* = 69)	10 (5–15) (*N* = 45)	18 (14–21) (*N* = 24)	<0.0001	0.0010

* *P-values based on Mann-Whitney, Chi^2^-Test or Fisher exact test as appropriate*.

** *P-values adjusted for age in a logistic regression*.

*ICU, intensive care unit; Covid-19, Corona virus disease 2019; No. patients, number of patients; BMI, body mass index; SOFA, Sepsis-related organ failure assessment score. Data are shown as median and interquartile range (25%-75%) or absolute numbers and percentage of patients, respectively. The data represent the analysis of 106 patients, unless otherwise specified via the n-number in the respective row*.

### Laboratory Findings

Laboratory findings are presented in Table 2. Patients who survived ICU treatment had lower levels of inflammatory markers on admission and during the course of therapy. A near three-fold difference in interleukin-6 (IL-6) was present between survivors and non-survivors at the time of ICU admission. 58.3% percent of the non-survivors had IL-6 levels >400 pg/ml. Bacterial specimens were found in 12.3% of the patients with no significantly differences between survivors or non-survivors. Nevertheless, a high percentage was already treated with antibiotics prior to ICU admission.

**Table 2:** Laboratory and microbiological findings.

	**All patients** **(*N* = 106)**	**Survivors** **(*N* = 69)**	**Non-survivors** **(*N* = 37)**	*P*-value^*^	**Adjusted** *p*-value^**^
**Laboratory data**					
Lactate on Admission (mmol/l)	1.3 (0.9–1.8) (*N* = 72)	1.2 (0.9–1.4) (*N* = 44)	1.7 (1.3–3.2) (*N* = 28)	0.0002	0.0025
Ferritin (μg/l) on Admission	1,917 (1,310–3,166) (*N* = 26)	1,563 (1,013–2,453) (*N* = 17)	2,794 (1,483–3,487) (*N* = 9)	0.1693	
Highest D-dimers (mg/l) during ICU stay	5.7 (2.1–15.6) (*N* = 74)	4.4 (1.4–15.6) (*N* = 51)	7.1 (3.6–15.7) (*N* = 23)	0.0768	0.0502
**Infection analyses**					
IL-6 (pg/ml) on admission	236.0 (80.3–608.0) (*N* = 64)	146 (49.8–374.5) (*N* = 40)	501.5 (236.0–1,019.5) (*N* = 24)	0.0004	0.0985
IL-6 > 400 pg/ml on Admission- No. patients (%)	23 (35.9)	9 (22.5)	14 (58.3)	0.0038	0.0046
IL-6 (pg/ml) at discharge or death	47 (18.8–447.5) (*N* = 72)	22.8 (11.0–44.8) (*N* = 43)	550.0 (200.0–2,957.0) (*N* = 29)	<0.0001	0.0440
White blood cell count (n^*^1000/μl) on admission	9.2 (6.3–11.8) (*N* = 104)	8.1 (5.6–11.3) (*N* = 67)	10.0 (7.4–12.7) (*N* = 37)	0.0111	0.0290
Lymphocyte count (n^*^1000/μl) at discharge or death	1.5 (0.8–8.4) (*N* = 76)	1.5 (0.8–9.0) (*N* = 53)	1.6 (0.8–6.0) (*N* = 23)	0.4386	0.1760
PCT (ng/ml) on admission	0.5 (0.3–2.0) (*N* = 99)	0.5 (0.2–0.9) (*N* = 65)	1.3 (0.5–5.5) (*N* = 34)	0.0029	0.1001
PCT (ng/ml) at discharge or death	0.8 (0.1–4.1) (*N* = 80)	0.2 (0.1–0.8) (*N* = 48)	3.9 (1.6–7.4) (*N* = 32)	<0.0001	0.0273
Pos. bacterial culture (all sources of culture)—no. patients (%)	13 (12.3)	5 (7.3)	8 (21.6)	0.0582	0.0741
Antibiotic treatment no. patients (%)	57 (64.8) (*N* = 88)	31 (56.4) (*N* = 55)	26 (78.8) (*N* = 33)	0.0397	
Antiviral therapy—no. patients (%)	26 (24.5)	17 (24.6)	9 (24.3)	0.9715	0.8392

* *P-values based on Mann-Whitney,Chi^2^-Test or Fisher exact test as appropriate*.

** *P-values adjusted for age in a logistic regression*.

*IL-6, interleukin-6; PCT, procalcitonin; No. patients, number of patients*.

*Data are shown as median and interquartile range (25%-75%) or absolute numbers and percentage of patients, respectively. The data represent the analysis of 106 patients, unless otherwise specified via the n-number in the respective row*.

### Respiratory Support

The median arterial oxygenation index (P_a_O_2_/F_i_O_2_) at the time of admission was 120 (IQR 88–164), indicating moderate to severe ARDS in the majority of patients. Overall, 55.6% had a moderate ARDS at admission; 35.8% of all patients and 63.8% of the non-survivors already suffered from a severe ARDS (P_a_O_2_/F_i_O_2_ < 100) at the time of ICU admission. Pulmonary gas exchange worsened in both populations. Prone positioning was performed in 78.9% of the cases. However, comparing the P_a_O_2_ at the time of ICU discharge or death, respectively, there was no significant difference. Median duration of mechanical ventilation was 9 (IQR 5.5–15.5) days and not significantly different between survivors and non-survivors. The same applies to lung mechanics or radiographic findings (Table 3). Chest X-ray pathologies were relatively minor compared to the degree of hypoxemia at admission. While deteriorating during the course of therapy, RALE scores were never significantly different between survivors and non-survivors. Moreover, RALE scores recovered in both groups toward the end of therapy.

**Table 3:** Characteristics of pulmonary function and outcome.

	**All patients** **(*N* = 106)**	**Survivors** **(*N* = 69)**	**Non-survivors** **(*N* = 37)**	*P*-value^*^	**Adjusted** p-value^**^
**Pulmonary gas exchange (on admission)**					
P_a_O_2_/F_i_O_2_	120 (88–164) (*N* = 83)	121 (88–167) (*N* = 56)	120 (88–156) (*N* = 27)	0.8269	0.5717
P_a_O_2_ (mmHg)	74.1 (61.0–90.0) (*N* = 103)	76.0 (61.0–88.1) (*N* = 69)	67.4 (59.4–104.0) (*N* = 34)	0.6259	0.2783
P_a_CO_2_ (mmHg)	39.0 (34.2–47.5) (*N* = 104)	38.1 (33–43.6) (*N* = 69)	44.8 (37–49.4) (*N* = 34)	0.0072	0.0287
s_a_O_2_ (%)	94.0 (91.1–97.1) (*N* = 102)	94.6 (91.7–97.0) (*N* = 68)	93.6 (89.0–98.0) (*N* = 34)	0.8230	0.1693
Lung compliance (ml/cmH_2_O)	43.1 (32.0–59.8) (*N* = 42)	43.2 (36.4–55.4) (*N* = 21)	41.2 (30.7–59.8) (*N* = 21)	0.6781	0.7680
RALE score	12.0 (5.5–28.5) (*N* = 28)	12.5 (8.0–28.0) (*N* = 18)	8.5 (4.0–29.0) (*N* = 10)	0.3368	0.9313
**Pulmonary gas exchange (during ICU stay)**					
Lowest P_a_O_2_/F_i_O_2_	100. (75–131.) (*N* = 61)	110 (81–142) (*N* = 42)	89 (60–111) (*N* = 19)	0.0437	0.0256
Lowest p_a_O_2_ (mmHg)	57 (49.8–66.5) (*N* = 72)	61.9 (53.8–68.0) (*N* = 46)	51.7 (45.0–59.0) (*N* = 26)	0.0082	0.0064
P_a_O_2_ (mmHg)—at ICU-Discharge/Death	73.0 (66.0–89.8) (*N* = 95)	73.0 (67.0–92.0) (*N* = 62)	75.0 (66.0–86.5) (*N* = 33)	0.7337	0.5492
Highest p_a_CO_2_ (mmHg)	56.0 (45.0–72.0) (*N* = 98)	52.0 (41.0–62.8) (*N* = 67)	71.4 (53.1–81.5) (*N* = 31)	0.0005	0.0045
RALE score day 7	16 (5–36) (*N* = 27)	16 (7–36) (*N* = 17)	13 (2–33) (*N* = 10)	0.4204	0.6911
RALE score at time of discharge/death	6 (3–18) (*N* = 28)	7 (4–12) (*N* = 18)	4.5 (2.0–33.0) (*N* = 10)	0.8101	
**Mechanical ventilation**					
Highest F_i_O_2_ (%)	90 (65.0–100) (*N* = 103)	85 (60–100) (*N* = 67)	100 (90–100) (*N* = 36)	0.0012	0.0018
Highest peep (cmH_2_O)	15 (11–16) (*N* = 95)	12 (10–15) (*N* = 59)	15 (14–17) (*N* = 36)	0.0016	0.0058
Highest P_Plat_ (cmH_2_O)	31 (26–34) (*N* = 92)	29.5 (24.0–33) (*N* = 58)	32 (31–36) (*N* = 34)	0.0013	0.0054
Prone positioning—no. patients (%)	76 (71.7) (*N* = 104)	45 (65.2) (*N* = 69)	31 (88.6) (*N* = 35)	0.0111	
**ECMO (*****N*** **=** **17)**					
Age	58 (51–63)	50 (47–52)	62 (57–67)	0.0137	
**Mode—no. patients (%)**					
vvECMO	17 (16.0)	6 (8.7)	11 (29.7)		
vvaECMO	2 (1.9)	0 (0.0)	2 (5.4)		
P_a_O_2_/F_i_O_2_–prior to ECMO start	58 (51–66)	58.0 (56.3–69)	59.6 (55–80)	0.8548	
P_a_CO_2_–prior to ECMO start	70.5 (60.5–77.7)	71.3 (60.5–77.7)	67.3 (60.5–76.5)	0.7963	
Duration—hours	164.5 (126.7–369.3)	164.5 (126.7–225.4)	217.7 (126.5–444.6)	0.6605	
Duration mechanical ventilation prior to ECMO—days	2 (1–6)	1.5 (1.0–2.0)	5 (2–6)	0.0961	
Survival (%)	35.3 (95%-CI 17.3–58.7)				
**Outcome**					
Duration of ICU treatment–days	11 (7–19) (*N* = 102)	15 (7–20) (*N* = 67)	9 (6.5–12) (*N* = 35)	0.0540	
Duration of mechanical ventilation—days	9 (4.5–15.5) (*N* = 100)	9 (4–17) (*N* = 65)	9 (5–15) (*N* = 35)	0.6795	
Survival—(%)	65 (95%-CI 55.6–73.5)				

* *P-values based on Mann-Whitney,Chi^2^-Test or Fisher exact test as appropriate*.

** *P-values adjusted for age in a logistic regression*.

*P_a_O_2_, arterial partial pressure of oxygen; F_i_O_2_, fraction of inspired oxygen; s_a_O_2_, arterial saturation of hemoglobin; PEEP, positive end-expiratory pressure, P_Plat_, plateau airway pressure; SOFA, sepsis-related organ failure assessment score; vvECMO, venovenous extracorporeal membrane oxygenation; vaECMO, venoarterial extracorporeal membrane oxygenation; ICU, intensive care unit; No. patients, number of patients*.

*Data are shown as median and interquartile range (25%-75%) or absolute numbers and percentage of patients, respectively. The data represent the analysis of 106 patients, unless otherwise specified via the n-number in the respective row*.

### Extracorporeal Membrane Oxygenation (ECMO)

Venovenous (vv) ECMO was utilized in 16.3% (*n* = 17) of the patients with a median age of 58 (IQR 51–63) years. Two patients received venoveno-arterial (vva) support due to acute cor pulmonale. ECMO patients had been on mechanical ventilation for a median of two (IQR 1–6) days. In three quarters of all cases, the use of ECMO was indicated due to refractory hypoxemia. Median P_a_O_2_/F_i_O_2_ at the time of ECMO commencement was 58 (IQR 51–66). Six patients (35.3%; 95%-CI 17.3–58.7) survived until ICU discharge.

### Antiviral Therapies

The majority of patients (75.5%) did not receive any antiviral or anti-inflammatory therapy, while 24.5% received adjunct therapies including oseltamvir (*n* = 10), remdesivir (*n* = 1), chloroquins (*n* = 10) or tocilizumab (*n* = 3). However, as the choice and duration of therapy was purely at the discretion of the attending physicians, a large number of heterogeneous substances and protocols were used. Hence, no further analyses were performed due to the small sample sizes.

## Discussion

The current study focused on the characteristics and outcome of COVID-19 patients admitted to the ICU in five German centers. Our study population mainly consisted of high-risk patients, where ARDS mortality rates of 40 to 46% can be expected (8). Half of our patients suffered from severe ARDS. Major findings include the identification of age, diabetes mellitus and higher SOFA scores on admission as factors associated with non-survival during ICU treatment. Furthermore, our observations indicate that standard ARDS treatment resolves acute hypoxemia in the majority of cases.

The proportion of patients surviving ICU care was 65.0% with a corresponding 95% CI of 55.6–73.5. Survival rates of ICU patients varied substantially between previous studies and different countries, for example, between 22 to 84% in China (9–12), 50% in Seattle (13), 33% in Washington State (14) and 61% in New York (15). In a retrospective cohort study from Italy only 46.6% of the patients requiring hospital admission survived (16). The ICNARC currently reports a survival of 60% in intensive care from the United Kingdom (17). A recent analysis of COVID-19 patients via the claims of the German Local Health Care Funds revealed an overall mortality of 22% and a mortality of 53% in patients requiring invasive ventilation. However, ARDS subtypes were not classified and risk factors of non-survival were not identified (18). Differences between countries may be due to variations in patient characteristics, as well as ICU admission criteria, criteria for ECMO, or availability of ICU capacities. All of the participating hospitals had sufficient resources to provide the best available standard care at any time. The workforce on the ICU of the participating hospitals was actually increased to counteract the big challenges associated with COVID-19, including a high number of patients requiring prone positioning, as well as time and effort associated with the use of personal protective equipment.

Advanced age has been uniformly reported as a risk factor for severe disease (12, 19) and was also associated with a worse outcome in our study. Diabetes mellitus was also reported as a factor associated with death from COVID-19 in critically ill in New York City and Lombardy (15, 16). It was associated with an approximately three-fold increased risk of death in our study. Arterial hypertension on the other hand was the most frequent comorbidity. Nevertheless its presence was not associated with a worse outcome and likely only represents the overall disease frequency (20). Although previously reported as a predictor of sepsis mortality (21), lymphocytopenia was not a distinctive feature in our ICU population. We did observe differences in SOFA scores and IL-6. IL-6 is perceived to be the central mediator of a cytokine release syndrome (22) and survivors had significantly lower IL-6 levels at the time of ICU admission. In this regard, preliminary data indicate that the administration of dexamethasone could improve survival in patients receiving respiratory support (23). Nevertheless, in our study treatment protocols for the use of glucocorticoids were not defined and dexamethasone was not utilized in any of the patients. Moreover, due to the small sample size, no multivariable prediction model to identify potential predictors of survival could be build.

The standards of ARDS treatment consist of prone positioning and protective mechanical ventilation with higher PEEP levels. All centers adhered to these guideline recommended therapies (24), although P_Plat_ values indicate difficulties in maintaining lung protective ventilation at all times. Both survivors and non-survivors had worsening lung injury during the course of treatment with a high percentage of prone positioning. Patients dying during ICU treatment suffered from a worse pulmonary function at time of ICU admission, however, interestingly the duration of mechanical ventilation was not significantly different to patients surviving ICU care. Furthermore, p_a_O_2_ values do not indicate hypoxemia at the time of death. The same applies to the RALE score or lung compliance, emphasizing that radiographic findings and lung mechanics often do not match the severity of disease (3). Antiviral or anti-inflammatory treatments were only utilized in a minority of the patients. The use of remdesivir was recently associated with faster COVID-19 recovery times, whereupon beneficial effects could not be shown in patients receiving mechanical ventilation or ECMO (25). In our cohort, approximately one fourth received antiviral treatment, whereas no significant difference in survival was observed.

Seventeen patients (16.3%) received vvECMO therapy. The overall rate of vvECMO treatment was higher compared to what has been reported from China (11, 12), the United States (15) and Italy (26). German Local Health Care Fund data recorded ECMO treatment in 7% of all ventilated patients in 920 German hospitals (18). The high ECMO rate in our study population emphasizes the severity of disease and that mainly specialized centers participated in the study. Nevertheless, the survival rate was lower in these patients and worse compared to other causes of ARDS.

Taken together, standard ARDS treatment according to published guidelines resolved acute hypoxemia in the majority of cases. Advanced age and diabetes mellitus increased the risk of non-survival. ICU triage with population-level decision making was not necessary and sufficient ICU equipment and personnel resources were available at any time. If the number of COVID-19 ICU patients re-increases, standard ARDS treatment provides a strong basis to ensure a good outcome in critically ill COVID-19 ARDS patients.

## Data Availability Statement

The raw data supporting the conclusions of this article will be made available by the authors, without undue reservation.

## Ethics Statement

The studies involving human participants were reviewed and approved by University of Würzburg and Frankfurt, as well as the medical association of Bavaria ethics board (Aschaffenburg) and Hessen (Offenbach, Kassel). Written informed consent for participation was not required for this study in accordance with the national legislation and the institutional requirements.

## Author Contributions

JH, EA, KZ, and CL contributed substantially to the conception and design of the study, the acquisition, analysis, interpretation of the data, and drafted the article. PM, QN, PHel, MS, PU-P, AS, IT, CaR, ChR, MK, JS, and DG-S contributed substantially to the acquisition and analysis of the data. PK, AB, ToS, BS, DW, HK, TW, SF, GE, RM, HM, and YZ contributed substantially to the acquisition of data, the interpretation of the data and provided critical revision of the article. ThS contributed substantially to the critical revision of the article. PHeu contributed substantially to the analysis, interpretation of the data and provided critical revision of the article. VR contributed substantially to the analysis of the data. All authors contributed to the article and approved the submitted version.

## Conflict of Interest

PHeu reports grants from German Ministry of Research and Education, German Research Foundation, European Union, Charité—Universitätsmedizin Berlin, Berlin Chamber of Physicians, German Parkinson Society, University Hospital Würzburg, Robert Koch Institute, German Heart Foundation, Federal Joint Committee (G-BA) within the Innovationfond, University Hospital Heidelberg (within RASUNOA-prime; supported by an unrestricted research grant to the University Hospital Heidelberg from Bayer, BMS, Boehringer-Ingelheim, Daiichi Sankyo), Charité—Universitätsmedizin Berlin (within Mondafis; supported by an unrestricted research grant to the Charité from Bayer), University Göttingen (within FIND-AF randomized; supported by an unrestricted research grant to the University Göttingen from Boehringer-Ingelheim), outside the submitted work. SF reports grants from DFG, BMBF, grants and personal fees from Abiomed, Amgen, Akzea, AstraZeneca, Bayer, Berlin-Chemie, Braun, Bristol-Myers Squibb, Boehringer, Daiichi Sankyo, MSD, Novartis, Pfizer, Sanofi-Aventis, Servier, Siemens, Zoll, outside the submitted work. GE reports grants and personal fees from Bayer, grants and personal fees from Novartis, grants and personal fees from Vifor Pharma Deutschland GmbH, outside the submitted work; PK reports other from FreseniusKabi, personal fees from BBraun, grants, personal fees and other from TEVARatiopharm, other from CSL Behring, other from Pajunk, other from APEPTICO Forschung und Entwicklung GmbH, outside the submitted work; KZ reports personal fees from Aesculap Akademie GmbH, personal fees from Affinites Sante, grants from Ashai Kasai Pharma, grants and personal fees from B. Braun AG, grants and personal fees from B. Braun Avitum AG, personal fees from Bayer AG, grants from Biotest AG, personal fees from Christian Doppler Stiftung, grants and personal fees from CSL Behring GmbH, personal fees from Cyto Sorbents GmbH, personal fees from Edward Lifescience Corporation, personal fees from Executive Insight AG, personal fees from Fresenius Kabi GmbH, personal fees from Fresenius Medical Care, personal fees from Haemonetics Corporation, personal fees from Hartmannbund Landesverband, personal fees from Health Adcances GmbH, personal fees from Heinen + Löwenstein GmbH, personal fees from Hexal AG, grants from INC Research, personal fees from Johnson and Johnson, personal fees from Josef Gassner, personal fees from Maquet GmbH, personal fees from Markus Lücke Kongress Organization, personal fees from Masimo International, personal fees from med Update GmbH, personal fees from Medizin and Markt Gesundheitswerk, personal fees from MSD Sharp and Dohme GmbH, personal fees from Nordic Group, personal fees from Nordic Pharma, grants from Novo Nordisc Pharma GmbH, grants from Pfizer Pharma GmbH, personal fees from Pharmacosmos, personal fees from Ratiopharm GmbH, personal fees from Salvia Medical GmbH, personal fees from Schering Stiftung, personal fees from Schöchl Medical Österreich, personal fees from Serumwerke, personal fees from Verlag für Printmedien und PR, Forum Sanitas, grants and personal fees from Vifor Pharma GmbH, personal fees from Wellington, personal fees from Werfen, outside the submitted work; HK served as a speaker and/or an Advisory Board Member for AbbVie, BMS, Gilead, Hexal, Janssen, MSD, Pfizer, ViiV and has received research funding from AbbVie, Arrowhaed, BMS, Gilead, Janssen, MSD, Novartis, German Liver Foundation, Hector Foundation, Virtual University of Bavaria, Federal Ministry of Education and Research, outside the submitted work. The remaining authors declare that the research was conducted in the absence of any commercial or financial relationships that could be construed as a potential conflict of interest.

## Abbreviations

COVID-19: coronavirus disease-19
SARS-CoV-2: severe acute respiratory syndrome coronavirus 2
ARDS: acute respiratory distress syndrome
ICU: intensive care unit
vvECMO: venovenous extracorporeal membrane oxygenation
RT-PCR: real-time reverse transcriptase polymerase chain reaction
SOFA: sepsis-related organ failure assessment
F_i_O_2_: fraction of inspired oxygen
p_a_O_2_/F_i_O_2_: arterial partial pressure of oxygen/fraction of inspired oxygen oxygenation index
PEEP: positive end-expiratory pressure
P_Plat_: maximum airway plateau pressure
P_mean_: mean airway pressure
RALE: radiographic assessment of lung edema
IQR: interquartile range (25–75%)
PCT: procalcitonin
IL-6: interleukin 6
VTE: venous thromboembolism.
