# Supplementary material for: COVID-19 Induced Acute Respiratory Distress Syndrome—A Multicenter Observational Study
Source: Front Med (Lausanne). 2020 Dec 18;7:599533. doi: 10.3389/fmed.2020.599533 (PMC7775385; doi:10.3389/fmed.2020.599533)
Supplement: Supplementary file 1 [file Table_1.docx]

# Table S1. Patients with Severe ARDS (p_a_O_2_/F_i_O_2_<100)

| Characteristic | All patients  (N=57) | Survivors  (N=34) | Non-Survivors  (N=23) | P-Value |
| --- | --- | --- | --- | --- |
| *Demographics* |  |  |  |  |
| Age (years) | 61 (54 – 74) | 58 (52 – 70) | 66 (57 – 79) | 0.0695 |
| SOFA at time of ICU Admission | 11 (4 – 16)  (N=41) | 6.5 (4.0 – 11.0)  (N=22) | 15.0 (11.0 – 16).0  (N=19) | 0.0006 |
| *Infection Analyses* |  |  |  |  |
| IL-6 [pg/ml]  on Admission | 352.5 (159.0 – 1257.0)  (N=34) | 212.0 (83.0- – 733)  (N=19) | 531.0 (184.0 – 2087.0)  (N=15) | 0.1500 |
| Antiviral Therapy – No. patients (%) | 17 (29.8) | 8 (23.5) | 9 (39.1) | 0.2066 |
| *Pulmonary Gas Exchange (on Admission)* |  |  |  |  |
| P_a_O_2_/F_i_O_2_ | 90.0 (69.5 – 150.0)  (N=47) | 90.0 (69.0 – 140.0)  (N=29) | 91.0 (78.0 – 150.0)  (N=18) | 0.6300 |
| P_a_O_2_ [mmHg] | 76.1 (61.0 – 87.6)  (N=54) | 75.6(61.0 – 86.0)  (N=34) | 76.6 (63.1 – 125)  (N=20) | 0.1705 |
| P_a_CO_2_ [mmHg] | 40.4 (35.0 – 48.0)  (N=55) | 39.3 (33.0 – 43.6)  (N=34) | 42.6 (37.0 – 49.0)  (N=21) | 0.1052 |
| Lowest P_a_O_2_/F_i_O_2_ | 78.0 (61.5-98.0)  (N=44) | 85.5 (67.0 – 107.0)  (N=26) | 66.5 (51.0 – 92.0)  (N=18) | 0.0696 |
| Highest P_a_CO_2_ [mmHg] | 62.5 (50.0 – 76.8)  (N=50) | 57.2 (44.9 – 69.2)  (N=32) | 74.0 (62.2 – 81.5)  (N=18) | 0.0044 |
| Highest PEEP [cmH_2_O] | 15 (13 – 16)  (N=53) | 14 (12 – 15)  (N=30) | 16 (15 – 18)  (N=23) | 0.0019 |
|  |  |  |  |  |
| Prone positioning - No. patients (%) | 44 (80.0) | 24 (70.6) | 20 (95.2) | 0.0264 |
| ECMO – No. patients (%) | 15 (26.3) | 5 (14.7) | 10 (43.5) | 0.0155 |
| *Outcome* |  |  |  |  |
| Duration of ICU treatment – days | 14 (9 – 19)  (N=54) | 16 (9 – 21)  (N=33) | 11 (9 – 18)  (N=21) | 0.2067 |
| Duration of Mechanical Ventilation – days | 9 (5 – 18)  (N=52) | 12 (5 – 18)  (N=31) | 9 (5 – 14)  (N=21) | 0.5750 |
| Survival – [%] | 59.7 (95%-CI 46.7-71.4) |  |  |  |

Abbreviations: P_a_O_2_: arterial partial pressure of oxygen; F_i_O_2_: fraction of inspired oxygen; ECMO: extracorporeal membrane oxygenation; ICU: intensive care unit; No. patients: number of patients

Data are shown as median and interquartile range (25%-75%) or absolute numbers and percentage of patients, respectively. The data represent the analysis of 56 patients, unless otherwise specified via the n-number in the respective row.
